# *Hominis Placenta* facilitates hair re-growth by upregulating cellular proliferation and expression of fibroblast growth factor-7

**DOI:** 10.1186/s12906-016-1180-3

**Published:** 2016-07-07

**Authors:** Hyung-Sik Seo, Dong-Jin Lee, Jae-ho Chung, Chang-Hyun Lee, Ha Rim Kim, Jae Eun Kim, Byung Joo Kim, Myeong Ho Jung, Ki-Tae Ha, Han-Sol Jeong

**Affiliations:** Department of Ophthalmology, Otolaryngology and Dermatology, Korean Medicine Hospital, Pusan National University, Yangsan, Republic of Korea; Department of Clinical Medicine, Graduate School, Kyung Hee University, Seoul, Republic of Korea; Chung Jaeho Traditional Korean Medical Clinic, Seoul, Republic of Korea; Department of Anatomy, College of Korean Medicine, Woosuk University, Wanju, Republic of Korea; College of food & biotechnology, Woosuk University, Wanju, Republic of Korea; Department of Pathology, College of Korean Medicine, Dongguk University, Goyang, Republic of Korea; Division of Longevity and Biofunctional Medicine, School of Korean Medicine, Pusan National University, Yangsan, Republic of Korea; Healthy Aging Korean Medical Research Center, School of Korean Medicine, Pusan National University, Yangsan, Republic of Korea; Division of Applied Medicine, School of Korean Medicine, Pusan National University, Yangsan, Republic of Korea

**Keywords:** *Hominis Placenta*, Hair growth, Anagen, Bromodeoxyuridine, Fibroblast growth factor-7

## Abstract

**Background:**

*Hominis Placenta* (HP) known as a restorative medicine in Traditional Chinese Medicine (TCM), has been widely applied in the clinics of Korea and China as an anti-aging agent to enhance the regeneration of tissue. This study was conducted to investigate whether topical treatment of HP promotes hair regrowth in the animal model.

**Methods:**

The dorsal hairs of 8-week-old C57BL/6 mice were depilated to synchronize hair follicles to the anagen phase. HP was applied topically once a day for 15 days. Hair growth was evaluated visually and microscopically. The incorporation of bromodeoxyuridine (BrdU) and expression of proliferating cell nuclear antigen (PCNA), fibroblast growth factor-7 (FGF-7) in dorsal skin tissue was examined by immunohistochemical analysis. Reverse transcription polymerase chain reaction (RT-PCR) was used to measure the mRNA expression of FGF-7.

**Results:**

HP exhibited potent hair growth-promoting activity in C57BL/6 mice. Gross examination indicated that HP markedly increased hair regrowth as well as hair density and diameter. Histologic analysis showed that HP treatment enhanced the anagen induction of hair follicles.

Immunohistochemical analysis revealed that BrdU incorporation and the expressions of PCNA were increased by treatment of HP. HP treatment significantly increased the expression of FGF-7, which plays pivotal roles to maintain anagen phase both protein and mRNA levels.

**Conclusions:**

Taken together, our results indicate that HP has a potent hair growth-promoting activity; therefore, it may be a good candidate for the treatment of alopecia.

## Background

The number of patients with alopecia is constantly increasing because of the stressful modern lifestyle. In a vast majority of cases, hair loss is attributable to hormones and genetic factors [1, 2]; however, other factors, such as autoimmune diseases, medications, and physiologic and psychological stresses have been linked to hair loss [3–5]. These causative factors can alter the hair follicle cycle.

Humans have about 5 million hair follicles at birth and new follicles do not develop after birth [6]. Each hair follicle, which produce hair shafts, undergo successive cycles consisting of anagen (growing phase), catagen (transitional phase), and telogen (resting phase). This cycling occurs over the lifetime of the individual. Initiation and progression of the hair follicle cycle are determined by intercellular communications between the dermis and surface epithelium [7]. However, the complex molecular interactions between the cells of the hair follicle are not fully understood and the exact cause of alopecia has not been elucidated [8].

Androgenic alopecia (AA) which is the most common form of hair loss in men is characterized by thinning and visible loss of frontal hair. Under the influence of dihydrotestosterone (DHT), terminal hair follicles gradually change to miniaturized follicles. Hair follicular miniaturization arises as a consequence of progressive shortening of the duration of successive anagen [9]. Therefore, extension of anagen phase could be a good strategy for the treatment of hair loss such as AA.

*Hominis placenta* (HP) has been used in Korea and China to improve general well-being. Since the 1950s, HP has been used as therapeutic agent. By stimulation of liver regeneration, and controlling of endocrine system, HP has been applied to the liver diseases [10], and climacteric symptoms [11]. In addition, placenta extract was known to have antioxidant, anti-inflammatory, wound healing, and nerve growth-promoting effects [12–15]. Therefore, it is possible that the various effects of HP could influence the hair growth cycle.

One clinical study has already drawn attention to the hair-growth promoting effects of placental extract [16]. More recently, there was another report about hair growth promoting effects of cow placenta [17]. In this study, we investigated the hair growth-promoting effect of HP by measuring the cellular proliferation and expression of FGF-7.

## Methods

### Materials

Human placental extracts was provided from the Korean Pharmacopuncture Institute (Seoul, Korea). Primary antibodies specific for bromodeoxyuridine (BrdU), proliferating cell nuclear antigen (PCNA), fibroblast growth factor-7 (FGF-7), ß-actin were purchased from Santa Cruz Biotechnology, Inc. (Santa Cruz, CA, USA). Biotinylated goat anti-mouse immunoglobulin G (IgG) and avidin-biotin peroxidase complex were purchased from Vector Laboratories, Inc. (Burlingame, CA, USA). HRP-linked anti-mouse Ig G antibody was purchased from GE Healthcare Life Science (Pittsburgh, PA, USA). 3-3'diaminobenzidine, PBS solution, and hydrogen peroxide were purchased from Sigma-Aldrich Co. (Youngin, Korea). Trizol reagent and a reverse transcriptase-polymerase chain reaction (RT-PCR) kit were purchased from Invitrogen (Carlslab, CA, USA) and Bioneer Co. (Daejeon, Korea), respectively.

### Animals and in vivo hair growth activity

Seven-week-old male C57BL/6 mice were purchased from Samtaco Bio Korea, Ltd. (Osan, Korea) and allowed to adapt to the new environment for one week. The mice were housed in certified, standard laboratory cages and were provided with food and water *ad libitum* prior to the experiment. Amount of foods that consumed by one mouse per one week was about 42 g, and the amount of drinking water in the same condition was about 60 ml. Fifteen mice were divided into the following three groups (5 mice per group): normal saline-, 5 % minoxidil-, and HP-treated groups. The animal protocol used in this study was reviewed by the Pusan National University-Institutional Animal Care and Use Committee (PNU-IACUC) according to their ethical and scientific procedures and was approved (Approval Number PNU-2014-0581).

**Fig. 1:**
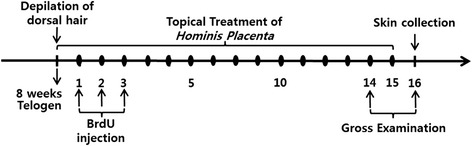
Experimental scheme. Dorsal hairs of 8-week-old C57BL/6 mice, which were in the telogen phase of the hair cycle, were depilated to synchronize anagen induction. The mice were divided into following three groups: group 1, normal saline-treated negative control; group 2, 5 % minoxidil-treated positive control; group 3, *Hominis Placenta* (HP)-treated experimental group (*n* = 5 per group). Each agent were topically applied from the 1st to the 15th day after hair depilation. Bromodeoxyuridine (BrdU) was administered twice a day on the 1st, 2nd, and the 3rd day after depilation. Depilated dorsal skin lesions were collected at the 16th day after depilation and stained with hematoxylin and eosin. ●, Topical treatment with normal saline, 5 % minoxidil, or HP

This study was designed according to the guidelines of the Korean Food and Drug Administration (KFDA) to evaluate hair growth promoting efficacy. The dorsal hairs of 8-week-old C57BL/6 mice, with hair follicles in the telogen phase of the hair growth cycle, were depilated to synchronize hair follicle growth to the anagen phase. One day after depilation, the mice were topically treated with normal saline, 5 % minoxidil, or HP once a day for 15 days (Fig. 1). Hair growth and thickness in the depilated dorsal skin lesions were measured by dermoscopy (Sometech, Inc., Seoul, Korea).

### Skin histology

C57BL/6 mice were euthanized at 16^th^ day after depilation. Dorsal depilated skin tissues were collected from the mice and fixed for 24 h at room temperature in Bouin’s solution. After dehydration, the skin tissues were embedded in paraffin, cut into 7-μm-thick sections, and placed onto glass slides. The slides were de-paraffinized with xylene and stained with hematoxylin and eosin (H&E). Processed skin tissues were examined by light microscopy (Carl Zeiss, Germany).

### Immunohistochemistry

BrdU, which is commonly used to label proliferating cells in tissue sections [18], was injected intraperitoneally at 50 μg/g b.w. twice daily for three consecutive days after hair depilation as described in a previous study [19]. Dorsal skin tissues were collected 16 days after the first treatment and subjected to immunostaining. Permeabilized tissue sections were incubated with the following antibodies by using the dilutions indicated in the manufacturer’s instructions: mouse anti-BrdU (1:200), anti-PCNA (1:1000), and anti-FGF-7 (1:200). After the primary antibody incubation, skin tissues were incubated with biotinylated goat anti-mouse IgG in a moisture chamber for 30 min. After washing, the tissues were incubated with HRP-conjugated avidin-biotin complex. After two more washing steps, the tissues were incubated with 30 mg of 3-3′diaminobenzidine dissolved in 150 mL of 0.1 M PBS solution for another 5 min, and then 0.005 % hydrogen peroxide was added for 15 min to develop the color. Immunoreactivity was examined by light microscopy.

### RT-PCR

Total RNA was isolated using TRIzol reagent, and complementary DNA (cDNA) was synthesized using AccuPower RT PreMix (Bioneer, Daejon, Korea) according to the manufacturer’s instructions. Specific DNA sequences were amplified with AccuPower PCR PreMix (Bioneer, Daejon, Korea). The oligonucleotide primer sequences were as follows: FGF-7, forward 5′-AGATCATGCTTCCACCTCGT-3′ and reverse 5′-TGGGTCCCTTTCACTTTGCC-3′; GAPDH, forward 5′-GGAGCCAAAAGGGTCATCAT-3′ and reverse 5′-GTGATGGCATGGACTGTGGT-3′. Amplified products were separated on 1.0 % agarose gels and analyzed under ultraviolet light. Images were captured using a GelDoc-It TS Imaging System (UVP, LLC, Upland, CA, USA).

### Western blot

To isolate proteins, skin tissues were lysed by RIPA buffer (0.1 % SDS, 1 % NP-40, 50 mM Tris, pH7.5, 150 mM NaCl, 50 mM NaF, 1 mM EDTA). Protein concentration of cell lysate was determined by Bradford method. After denaturation of protein at 100 °C for 5 min, 10 μg of proteins were loaded on the 10 % polyacrylamide gel, then separated by electrophoresis. After separation, the proteins were transferred to the polyvinylidene difluoride (PVDF) membrane. The membrane was blocked by 5 % non-fat milk in TBST for 1 h at room temperature. After blocking, the membrane was washed by TBST 3 times, then incubated with 1: 500 diluted mouse monoclonal anti-FGF7 antibody at 4 °C for overnight. The next day, the membrane was washed 3 times by TBST, then incubated with anti-mouse Ig G HRP-linked antibody for 1 h, at room temperature. After finishing another 3 times of washing, enhanced chemilumescent (ECL) substrates were added to the membrane for the detection of antibody. Images were captured by using Amersham Imager 600 (Pittsburgh, PA, USA).

### Statistical analyses

Data are expressed as the mean ± standard deviation (SD). Statistical differences between means were determined by one-way analysis of variance (ANOVA) for repeated measures. *P* values less than 0.05 were considered significant.

## Results

### HP promotes hair re-growth on the depilated skin lesions of C57BL/6 mice

**Fig. 2:**
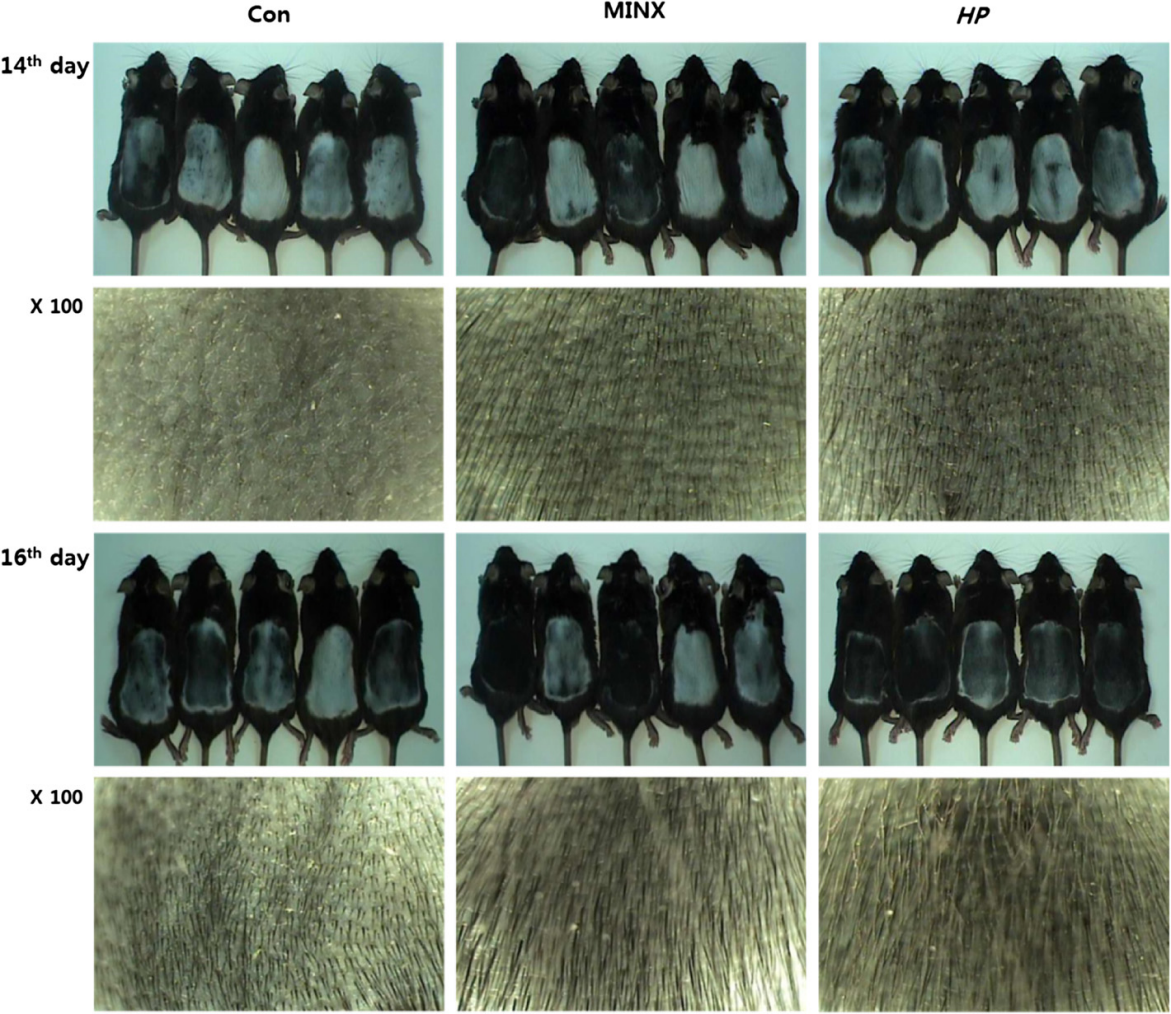
Gross observation of hair re-growth in C57BL/6 mice. At the 14th and 16th days after depilation, skin color and hair growth of the depilated skin lesions were observed. Each group of mice has been topically treated with normal saline or 5 % minoxidil or HP once a day for consecutive 15 days respectively. Depilated dorsal skin lesions were photographed at the 14th and 16th day after depilation. Hair regrowth, as well as skin darkness, was increased in mice treated with HP as compared to normal control mice

We tested the hair growth-promoting activity of HP. HP was applied topically daily onto the depilated dorsal lesions of C57BL/6 mice. Normal saline, 5 % minoxidil were used as the negative and positive control, respectively (Fig. 2, left and middle panel). HP promoted hair re-growth to a similar extent as minoxidil (Fig. 2, right panel). The skin color of HP-treated mice changed from pale to dark gray/black, indicating initiation of anagen.

### HP also increases hair density and thickness in C57BL/6 mice

**Fig. 3:**
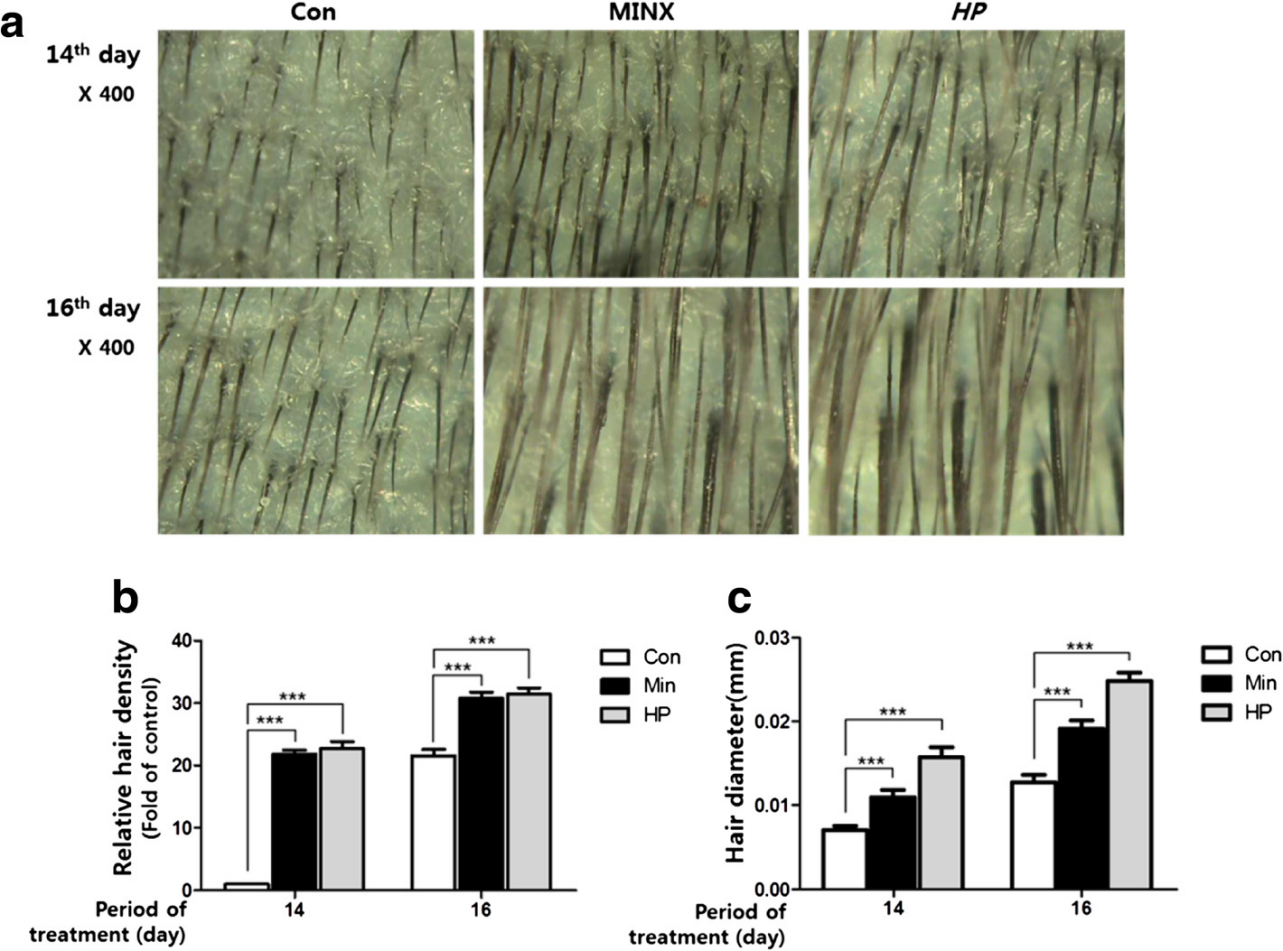
Dermoscopic observation of hair regrowth in C57BL/6 mice. Dorsal skins of mice were photographed by dermoscopy at the 14th and 16th day after depilation. Hair density was expressed as the number of hairs per unit area. Compared to the skin of the mice in the normal saline-treated control group (Con), the skin of the mice in the 5 % minoxidil (Min)- or HP-treated group had increased hair density and diameter (**a**). Hair density and hair diameter was greater in HP treated group than normal control group (**b**, **c**). Original Magnification: X100, X400. Values represent the mean ± standard deviation. ***P < 0.001, compared to control

Hair density and thickness were examined by dermoscopy. Figure 3a shows dermoscopic images of hair density and diameter at the 14^th^ and 16^th^ day after depilation. Hair density was higher in HP- or 5 % minoxidil-treated mice than in normal saline-treated mice. A significant difference in hair density was observed at day 14; however, this difference decreased by at day 16 (Fig. 3a, b). Hair diameter was also larger in the HP- and 5 % minoxidil-treated mice than in the mice treated with normal saline (Fig. 3a, c).

### HP enhanced the anagen induction and the cellular proliferation of hair follicle

**Fig. 4:**
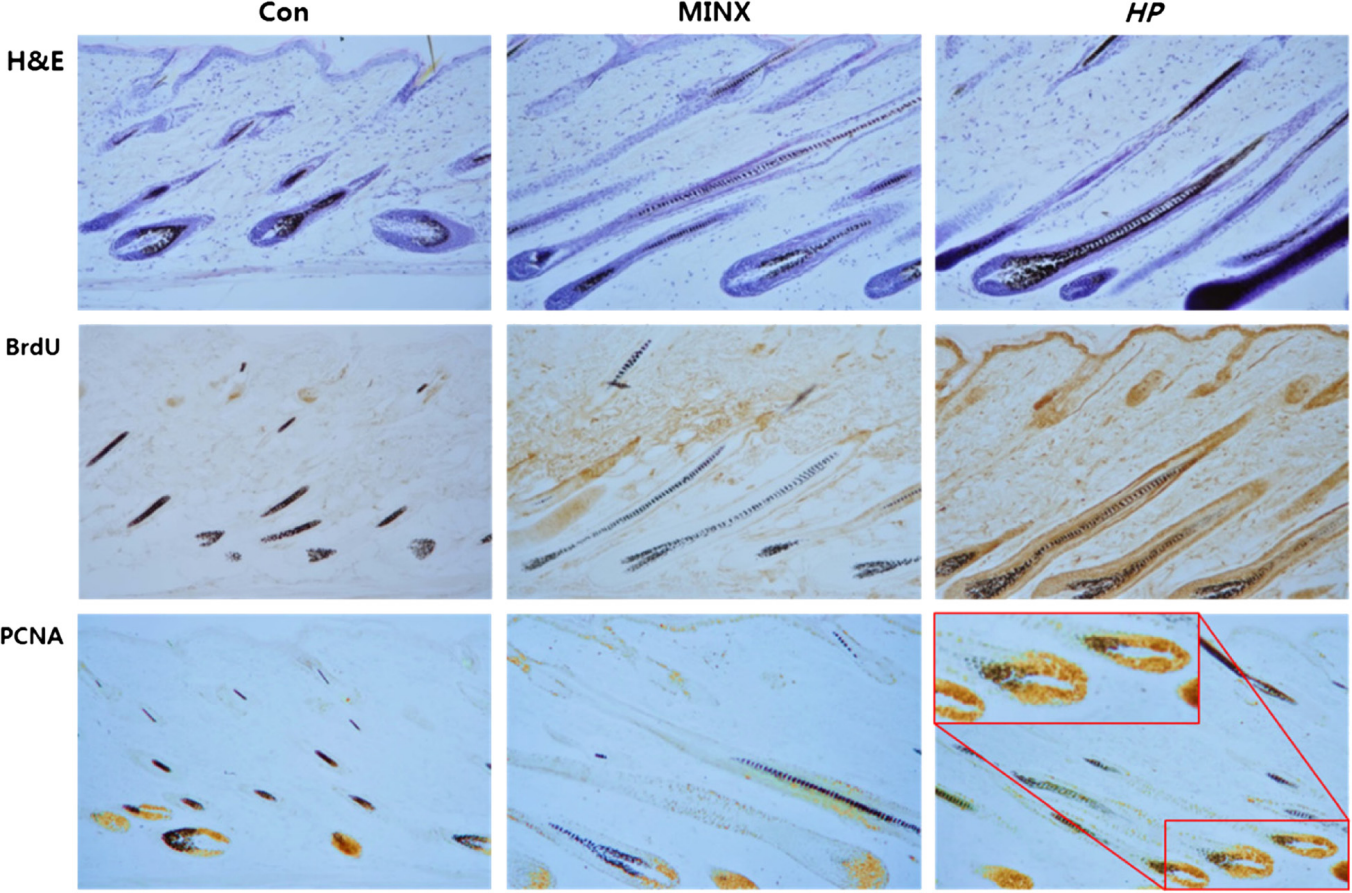
Microscopic observation of hair follicle in C57BL/6 mice. Histologic examination reveals hair follicle development in C57BL/6 mice treated with normal saline or MINX or HP for 15 days. Dorsal skin sections were stained with hematoxylin and eosin. Immunohistochemical staining were performed to examine the expression of BrdU and PCNA. MINX: minoxidil; *HP: Hominis placenta*; Original magnification: X100, large box: X200

Histological examination showed that hair follicles in the normal saline-treated mice resided in the dermis, indicating that they had not fully developed to anagen (Fig. 4, upper left). However, the hair shafts of follicles in the skin of HP-treated mice extended through the epidermis, indicating that they were fully developed to anagen IV phase (Fig. 4, upper right). Immunohistochemical analysis confirmed that BrdU incorporation was prominently increased by HP (Fig. 4, middle right). Strongly positive reactions against BrdU were observed in the bulge, inner and outer root sheath, panniculus carnosus, and epidermal epithelium, indicating that HP induced active cell division in the hair follicle. HP also increased the expression of PCNA in the hair bulb, and in the inner and outer root sheath (Fig. 4, lower right, large box).

### HP upregulates the expression of fibroblast growth factor-7 (FGF-7) involved in anagen development

**Fig. 5:**
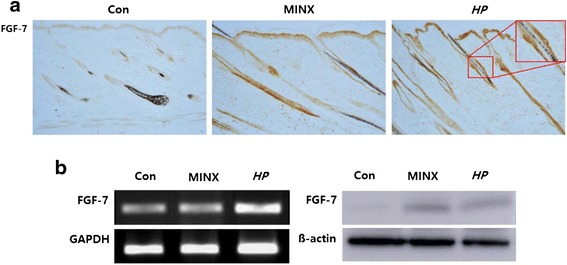
HP upregulates the expression of FGF-7 involved in anagen development Dorsal skins were collected from C57BL/6 mice treated with normal saline (Con), 5 % minoxidil (MINX), or HP for 15 days. Immunohistochemical analysis of hair follicles against FGF-7 was performed (**a**). Original magnification: X100, large box: X200. RT-PCR and western blot method was used to determine the expression of FGF7 at the mRNA and protein level (**b**)

HP increased the expression of FGF-7 in the epidermis, and inner and outer root sheath, to a degree comparable to minoxidil treatment (Fig. 5a, right, large box). HP also increased the mRNA and protein expression of FGF-7, key molecule in tissue repair and anagen induction in the dorsal skin lesions of C57BL/6 mice (Fig. 5b).

## Discussion

Placenta exerts critical roles in the development of fetus by exchanging nutrient, oxygen, waste products between mother and fetus, producing hormones which support the pregnancy. Placenta contains various bioactive substances including nucleic acid, amino acid [20]. Essential and non-essential amino acids contained in placenta were reported to be essential for the making fetal proteins [21]. Placenta has been used for the medical purpose since thousand years ago, in Eastern countries. In Traditional Chinese Medicine, human placenta (HP) has been used to enhance the vital essence [22]. Nowadays HP is considered as a source of cells with stem cell potential and could be able to applicate the tissue injuries [23]. Although a number of clinical studies about the beneficial effects of placenta extracts has been reported [24–27], the vast majority of the functional studies of the placental extracts was focused on it’s wound healing potential in the tissue repairing process [28–31]. Placenta extract was reported to increase the expression of bFGF and TGF-β1, two key factors involved in wound healing in the rat wound skin [14]. Aqueous extract of placenta was known to contain various peptides responsible for tissue regeneration [29]. Angiogenesis is a key process in the wound healing. New vessels deliver oxygen and nutrients to the injured lesions thereby facilitating tissue regeneration [32]. Angiogenic factor which suggested to be peptide was extracted from placenta [33]. Placenta extracts also induced nitric oxide (NO), a vasodilator in the mouse peritoneal macrophages [34]. More recently, placenta extract was also reported to inhibit Proteinase K, a microbial protease, thereby possibly prevent excessive proteolysis observed in bacterial infections [35].

Besides the wound healing, hair follicle cycle represents the other physiological regenerating event occurring in the skin. Growth factors involved in the wound healing are also involved in the hair follicle cycle [7]. Therefore, regenerative potential of placenta extract could also influence the hair cycle. Among the bioactive substance contained in placenta extract, several amino acids were known to positively influence hair growth. Arginine, a precursor of nitric oxide, plays an important role in skin angiogenesis [36]. Glutamine delivers sulphur which is necessary for hair growth [37]. Women with increased hair shedding responded to l-lysine and iron therapy [38].

Hair follicles undergo successive growth, regression, rest, and shedding over the life time. Hair follicular cells including mesenchymal cells of dermal papilla and overlying epithelial cells which is responsible for making hair shaft only proliferate during anagen phase [6]. Therefore, length and thickness of each hair shafts largely determined by the duration of anagen. Hair follicle miniaturization, one of the most characteristic features of androgenic alopecia, was due to the progressive shortening of anagen in response to DHT [2]. Therefore, a rational strategy for the treatment of androgenic alopecia is to inhibit DHT activity or to prolong the duration of the anagen phase. Transition from anagen to categen is regulated by various molecules released from hair follicular cells. Among them Fibroblast growth factor-7 (FGF-7) was known to play a pivotal role in the reentering of hair follicle to the next anagen phase [7, 39]. FGF-7, also known as keratinocyte growth factor, which plays important role in the regulation of proliferation in epithelial tissues [40], also protects the hair follicle from cytotoxic agents or UV irradiation [41, 42].

In this study, we found that HP treatment accelerated anagen induction in the hair follicles of C57BL/6 mice. Gross observation showed that the capacity of HP to enhance hair growth in C57BL/6 mice was comparable to that of minoxidil. Histological examination revealed that hair follicles in normal saline-treated mice were smaller and less developed than those in minoxidil- or HP-treated mice. This observation is consistent with the gross examination indicating that HP has the potential to enhance anagen induction. As expected, hair density and diameter were also increased by the treatment of HP.

To evaluate the effect of HP on hair matrix cell proliferation during anagen, we examined the pattern of BrdU incorporation and PCNA expression. High levels of BrdU incorporation were observed in the dermal papilla, outer layer of the hair shaft, and connective tissue. PCNA was highly expressed in the lower regions of the hair matrix, indicating that HP treatment induced DNA synthesis (Fig. 4, lower left). This finding is consistent with the previous observation that HP increases hair diameter, because hair thickness is proportional to the volume of the hair matrix.

It is well known that the hair cycle is regulated by complex epithelial-mesenchymal interactions. A number of growth factors in the dermal papilla and overlying epithelial cells are known to interact with each other during hair growth cycle progression [43]. FGF-7 plays a pivotal role on the hair follicular cycle [44, 45]. We found that FGF-7 expression was increased in the outer layer of the hair shaft and surface epithelium by the treatment of HP. Increased expression of FGF-7 in the skin tissues both mRNA and protein level, determined by RT-PCR and western blot respectively.

We showed that topical treatment with HP induced robust hair re-growth in C57BL/6 mice owing to proliferation of hair matrix cells and upregulation of FGF-7. This study helps to understand the basic mechanism through which HP acts on hair follicle development. In particular, increased expression of FGF-7 by HP explains it’s positive effects on the anagen induction.

## Conclusions

HP has a potent hair growth-promoting activity; therefore, it may be a good candidate for the treatment of alopecia.

## Abbreviations

AA, androgenic alopecia; BrdU, bromodeoxyuridine; DHT, dihydrotestosterone; ECL, enhanced chemilumescent; FGF-7, fibroblast growth factor-7; HP, *Hominis Placenta;* PCNA, proliferating cell nuclear antigen; PVDF, polyvinylidene difluoride; RT-PCR, reverse transcriptase-polymerase chain reaction
